# A Novel Highly Divergent Strain of Cell Fusing Agent Virus (CFAV) in Mosquitoes from the Brazilian Amazon Region

**DOI:** 10.3390/v10120666

**Published:** 2018-11-24

**Authors:** Licia Natal Fernandes, Thaís de Moura Coletti, Fred Julio Costa Monteiro, Marlisson Octavio da Silva Rego, Edcelha Soares D’Athaide Ribeiro, Geovani de Oliveira Ribeiro, Robson dos Santos Souza Marinho, Shirley Vasconcelos Komninakis, Steven S. Witkin, Xutao Deng, Eric Delwart, Ester Cerdeira Sabino, Élcio Leal, Antonio Charlys da Costa

**Affiliations:** 1 Laboratory of Protozoology, Institute of Tropical Medicine, University of São Paulo, São Paulo, SP 05403-000, Brazil; thaiscoletti@gmail.com; 2 Laboratory of Vectors, Superintendence for the Health Survaillance of Amapá, Macapá, AP 68905-230, Brazil; fredjulio@gmail.com (F.J.C.M.); farmarlisson@hotmail.com (M.O.d.S.R.); edcelhamanu@hotmail.com (E.S.D.R.); 3 Institute of Biologycal Sciences, Federal University of Pará, Belém, Pará 66075-000, Brazil; geovanioribeiro@gmail.com (G.d.O.R.); elcioleal@gmail.com (E.L.); 4 Laboratory of Retrovirology, Federal University of São Paulo, São Paulo, SP 04039-032, Brazil; robsonsantos@id.uff.br (R.d.S.S.M.); skomninakis@yahoo.com.br (S.V.K.); 5 Faculty of Medicine of ABC, Santo André, SP 09060-870, Brazil; 6 Department of Obstetrics and Gynecology, Weill Cornell Medicine, New York, NY 10065, USA; witkinss@gmail.com; 7 Institute of Tropical Medicine, University of São Paulo, São Paulo, SP 05403-000, Brazil; sabinoec@gmail.com; 8 Vitalant Research Institute, 270 Masonic Avenue, San Francisco, CA 94118-4417, USA; xdeng@vitalant.org (X.D.); eric.delwart@ucsf.edu (E.D.); 9 Department of Laboratory Medicine, University of California, San Francisco, CA 94143, USA; 10 Department of Infectious Diseases, Faculty of Medicine, University of São Paulo, São Paulo, SP 01246-903, Brazil

**Keywords:** cell fusing agent virus, Culex flavivirus, flavivirus, *Culex* sp., *Aedes aegypti*, mosquitoes, Amazon region

## Abstract

Classical insect-specific flaviviruses (cISFs) have been widely detected in different countries in the last decades. Here, we characterize the near full-length genomes of two cISFs detected in mosquitoes collected in the city of Macapá, state of Amapá, Amazon region of Brazil. A total of 105 pools of female mosquitos were analyzed by next-generation sequencing (NGS). Comparative genomics and phylogenetic analysis identified three strains of cell fusing agent virus (CFAV) and two of Culex flavivirus (CxFV). All sequences were obtained from pools of *Culex* sp., except for one sequence of CFAV detected in a pool of *Aedes aegypti*. Both CxFV strains are phylogenetically related to a strain isolated in 2012 in the Southeast region of Brazil. The CFAV strains are the first of this species to be identified in Brazil and one of them is highly divergent from other strains of CFAV that have been detected worldwide. In conclusion, CFAV and CxFV, circulate in mosquitoes in Brazil. One strain of CFAV is highly divergent from others previously described, suggesting that a novel strain of CFAV is present in this region.

## 1. Introduction

Insect-specific flaviviruses (ISFs), unable to replicate in mammalian cells, have been widely detected in mosquitoes from different countries in the last decades [1,2,3,4,5]. This group comprises the classical ISFs (cISFs), which are phylogenetically different from all other known flaviviruses [1].

The first cISF to be described was cell fusing agent virus (CFAV), which was isolated from an *Aedes aegypti* cell line [3,6] and subsequently detected in mosquitoes from Puerto Rico [7], Thailand [8,9], Indonesia [10], Mexico [11] and Kenya [12].

Apart from CFAV, many other cISFs, including Culex flavivirus (CxFV), Kamiti river virus (KRV) and Aedes flavivirus (AEFV), have been isolated and characterized [1,2]. Culex flavivirus is the most reported cISF to date. It has been found to infect different species of *Culex* mosquitoes in Japan and Indonesia [13], Guatemala [14], Mexico [15,16,17], the USA [18,19,20,21,22], Trinidad [18], Uganda [23], Brazil [24,25], China [26,27,28], Taiwan [29], Argentina [30], Kenya [12] and Myanmar [31].

There are a few reports on cISFs in Southeast Brazil. CxFV was first isolated in the city of São José do Rio Preto and a fragment of the *NS5* gene was sequenced [24]. More recently, the complete genome of this isolate was published [32]. In the city of São Paulo, CxFV and AEFV sequences of the *NS5* gene have also been detected in mosquitos [25]. To the best of our knowledge, no information is available on the occurrence of cISF in other regions of the country.

Despite the widespread occurrence of cISFs, there is little information regarding their frequency, distribution, host range and genetic diversity. Therefore, we performed a metagenomics survey in mosquitoes from the North of Brazil, a region with no previous information on cISF and where mosquitoes are highly abundant.

## 2. Materials and Methods

### 2.1. Location of Sample Collection

**Figure 1:**
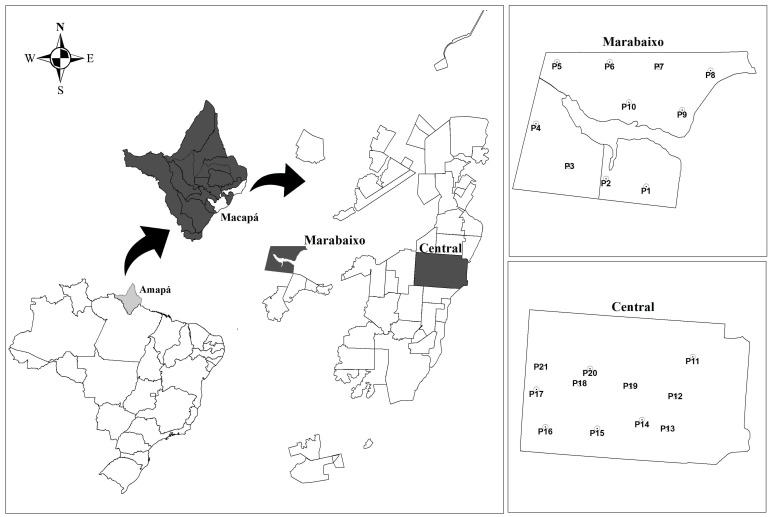
Location of the study area. From left to right: Map of Brazil highlighting the state of Amapá, map of Amapá highlighting the city of Macapá, map of Macapá highlighting the neighborhoods of Marabaixo and Central and maps of the two neighborhoods showing the 21 locations where mosquitoes were collected.

Mosquitoes (Diptera: Culicidae) were collected in the city of Macapá, Amapá (AP), Northern Brazil. Macapá is the largest city and also the capital of Amapá. It is located in the Amazon region. A population of 474,706 inhabitants was estimated in 2017 [33]. Collections of mosquitoes were performed in either residential or commercial properties at 21 points located in two neighborhoods, Central and Marabaixo (Figure 1). Central was the first neighborhood to be formed and consists of a commercial and administrative center. Its population of 17,798 inhabitants live in an area of 4.1 km^2^, with 4831 households [33]. Marabaixo is a non-official neighborhood and shows a lower degree of urbanization compared to Central. Coordinates of each point of collection were obtained using the Universal Transverse Mercator System (UTM), by means of Garmin Oregon 550 GPS (Garmin International, Inc., Olathe, Kansas, USA) and QGIS 3.0 software (QGIS^®^).

### 2.2. Collection and Identification of Mosquitoes

Insect collections were carried out twice a month during the morning (8 to 10 a.m.) from January to March 2017. Electric manual aspirators [34], Castro aspirators [35] and entomological nets were used to collect the mosquitoes inside and outside of residential and commercial properties.

The mosquitoes were transported to the laboratory, euthanized with ethyl acetate and morphologically identified using the dichotomous keys of Consoli and Lourenço-de-Oliveira [36]. After identification, mosquitoes had their wings and legs removed. Up to five females were grouped in pools according to their taxonomic category, place and date of collection. Mosquitoes were stored in a −80 °C freezer after identification.

A total of 127 pools of mosquitoes were obtained, 90 from Marabaixo and 37 from Central. A total of 105 of the 127 pools of mosquitoes were analyzed according to the following protocol.

### 2.3. Sample Processing and Next Generation Sequencing (NGS)

The protocol used to perform deep sequencing was a combination of several protocols normally applied to viral metagenomics and/or virus discovery [37], and has been partially described by da Costa et al. [38]. In summary, each mosquito pool was diluted in 900 µL of Hanks’ buffered salt solution (HBSS), added to a 2 mL impact-resistant tube containing lysing matrix C (MP Biomedicals, Waltham, MA, USA), and homogenized in a FastPrep-24 5G Homogenizer (MP Biomedicals, Waltham, MA, USA). The homogenized sample was centrifuged at 12,000 × *g* for 10 min, and approximately 300 µL of the supernatant was then filtrated through a 0.45 µm filter (Merck Millipore, Billerica, MA, USA) to remove eukaryotic and bacterial cell-sized particles. Approximately, 100 µL, roughly equivalent to one-fourth of the volume of the tube, of cold PEG-it Virus Precipitation Solution (System Biosciences, Palo Alto, CA, USA) was added to the obtained filtrate, the contents of the tube were gently mixed and then incubated at 4 °C for 24 h. The mixture was then centrifuged at 10,000× *g* for 30 min at 4 °C and the supernatant (~350 µL) was discarded. The viral particle-rich pellet was treated with a combination of nuclease enzymes (TURBO DNase and RNase Cocktail Enzyme Mix-Thermo Fischer Scientifc, Waltham, CA, USA; Baseline-ZERO DNase - Epicentre, Madison, WI, USA); Benzonase (Merck KGaA, Darmstadt, Germany); and RQ1 RNaseFree DNase and RNase A Solution (Promega, Madison, WI, USA) to digest free nucleic acids. The resulting mixture was subsequently incubated at 37 °C for 2 h. Viral nucleic acids were then extracted using ZR & ZR-96 Viral DNA/RNA Kit (Zymo Research, Irvine, CA, USA), according to the manufacturer’s protocol. The cDNA synthesis was performed with AMV Reverse transcriptase (Promega, Madison, WI, USA). A second strand of cDNA was synthesized using DNA Polymerase I Large (Klenow) Fragment (Promega, Madison, WI, USA). Subsequently, a Nextera XT Sample Preparation Kit (Illumina, San Diego, CA, USA) was used to construct a DNA library, identified using dual barcodes. Individual samples were then purified using the ProNex Size-Selective Purification System (Promega, Madison, WI, USA). For size range, Pippin Prep (Sage Science, Beverly, MA, USA) was used to select a 300 bp insert (range 200–400 bp). The library was deep-sequenced using the HiSeq 2500 Sequencer (Illumina, San Diego, CA, USA) with 126 bp ends. Bioinformatic analysis was performed according to the protocol previously described by Deng et al. [39]. The resulting singlets and contigs were analyzed using BLASTx to search for similarity to viral proteins in GenBank’s Virus RefSeq. The contigs were compared to the GenBank non-redundant nucleotide and protein database (BLASTn and BLASTx).

### 2.4. Phylogenetic Analyses

Based on the best hits of the Blast searches, the following genomes, listed by their GenBank numbers, were chosen. Near full-length sequences used to the inference of the genome tree: JX897904; GQ165808; KT726939; EU879060; JQ518484; HQ678513; JQ308187; FJ502995; AB262759; AB377213; FJ663034; AB701769; AB701775; AB701772; AB701768; AB701766; AB701773; JQ308188; JQ308190; AB981186; FJ644291; KC464457; KX652377; KX652376; KX652378; KX652375; HE574573; HE574574; KC505248; GQ165809; NC024299; GQ165810; NC001564; NC005064; KJ741266; KC181923; AB488408; KU201526; KP688057; KP688058; NC030290; JQ268258. Sequences used for the inference of the envelope (*E*) region tree (this region corresponds to the positions 920–2014 of the genome of the reference NC001564, Galveston): AB488425; AB813726; AB813727; AB813728; AB813729; AB813730; AB813732; AB813733; AB813734; AB813735; AB813737; AB813738; AB813739; AB813741; B813742; AB813743; AB813744; B813745; AB813749; AB813750; B813753; B813754; GQ165810; KJ476731; KJ741267; KU936054; M91671; MH237596; MH310082. These sequences were then aligned using Mafft software online [40]. To the inference of the *NS5* gene tree (this region corresponds to the positions 7463–9889 of the genome of the reference NC001564, Galveston), besides the sequences listed above the isolate KJ476731 was also used. Phylogenetic trees were reconstructed using the Maximum Likelihood approach, and branch support values were assessed using the Shimodaira-Hasegawa test. The genome tree was inferred using FastTree software [41] using the general time reversible (GTR) model and gamma distribution according to the likelihood ratio test (LRT) implemented in the jModeltest software [42]. We also used Bayesian Markov chain Monte Carlo method implemented in MrBayes version 3.2.3 [43] assuming GTR substitution model with gamma-distributed rate variation across sites and a proportion of invariable sites. Chains were run for 10 million generations, with the first 25% discarded as burn-in. Additional evolutionary analyses were conducted using MEGA7 [44]. Trees were edited and viewed with FigTree v1.4.2 (http://tree.bio.ed.ac.uk/software/figtree/).

## 3. Results

We processed a total of 105 pools of mosquitoes that were submitted to NGS. The main characteristics of pools in which near full-length flavivirus genome sequences were detected are shown in Table 1.

### 3.1. Phylogenetic Analysis of Genomes

**Figure 2:**
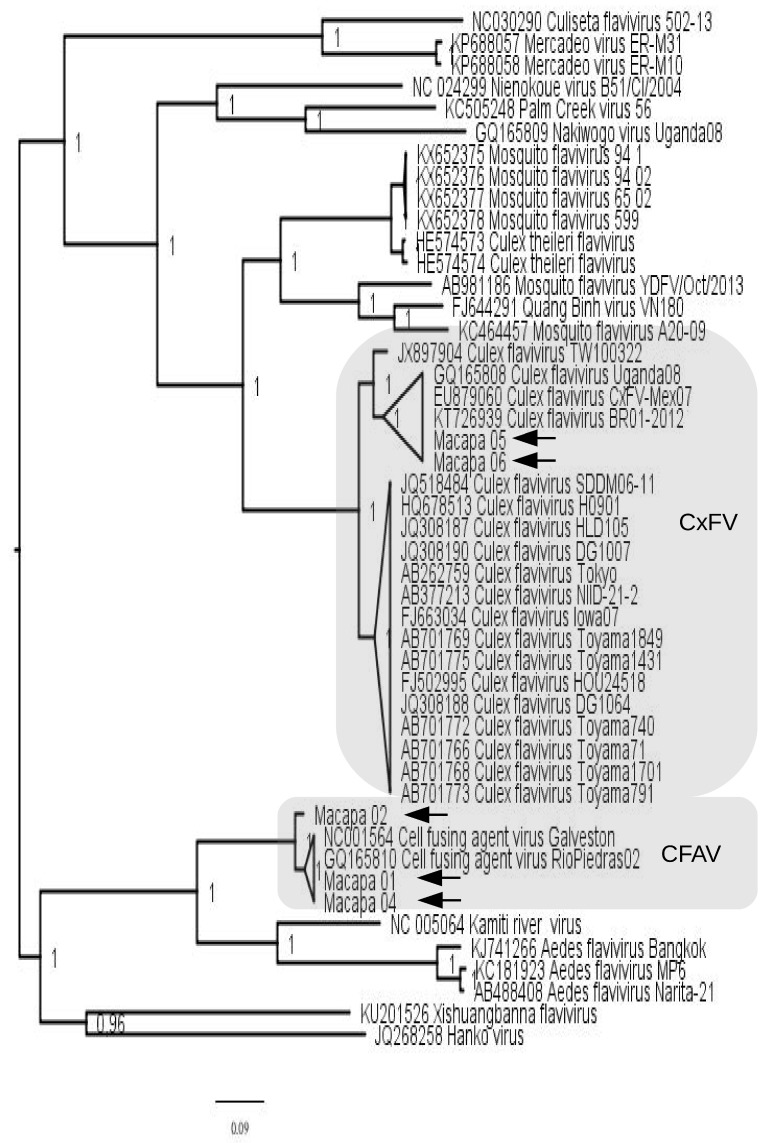
Maximum likelihood tree reconstructed using near full-length genomes of classical insect-specific flaviviruses (cISFs). The Brazilian strains described in this study are indicated by arrows. Values in the tree indicate the statistical support of each node, each of which was calculated using the approximate likelihood ratio test (aLRT). The topology shows that three strains (Macapá 01, 02 and 04) are closely related to cell fusing agent virus (CFAV) and the other two (Macapá 05 and 06) to Culex flavivirus (CxFV). The scale bar under the tree represents the nucleotide substitutions per site.

The near full-length genomes of the Brazilian strains were compared to cISFs available in the GenBank and a maximum likelihood tree was inferred (Figure 2). The tree shows that strains Macapá 01, Macapá 02 and Macapá 04 clustered in the clade formed by cell fusing agent virus (CFAV) isolates while the strains Macapá 05 and Macapá 06 clustered in the Culex flavivirus (CxFV) clade. The strains of CxFV from this study are in a phylogroup in which the basal strain was detected in Uganda (GQ165808), in 2008. Our CxFV strains are closely related to an isolate (KT726939) detected in the Southeast region of Brazil in 2012. In this clade there is also a Mexican strain isolated in 2007 (EU879060). Two strains of CFAV detected in this study (Macapá 01 and 04) cluster with the historical isolate GQ165810 (Rio Piedras02) from Puerto Rico identified in *Aedes aegypti*. Additionally, we found one CFAV strain (i.e., Macapá 02) divergent from all the other strains. The genetic distance based on the near full-length genomes between Macapá 02 and GQ165810 was 52% while the distance between GQ165810 and Macapá 01 was just 10% (Table S1). For this reason, a more detailed evolutionary analysis was performed with the CFAV strains.

Similarity (%) of the sequences of the strains Macapá 01 and 05 with sequences of flavivirus from GenBank are shown in Tables S2 and S3, respectively.

### 3.2. Phylogenetic Trees of CFAV Envelope (E) and NS5 Genes

**Figure 3:**
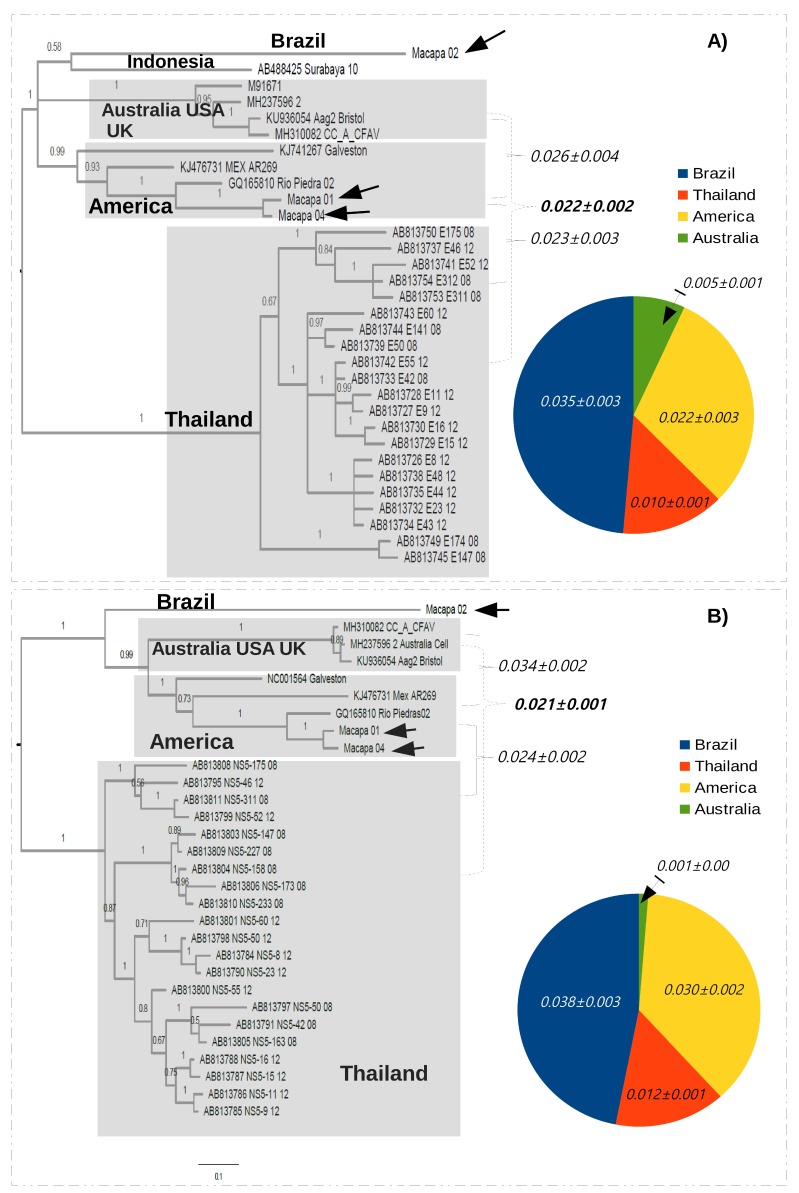
Bayesian trees reconstructed using the *E* and the *NS5* genes (**A** and **B**, respectively) of cell fusing agent virus (CFAV). The Brazilian strains described in the present study are indicated by arrows. Values on the trees indicate the statistical support (posterior probabilities) of each node. The topology shows three groups (gray areas) containing strains from certain geographical regions (i.e., Americas, Australia/USA/UK and Thailand). Braces indicate the genetic distances between phylogroups. The scale bar under the trees represents the nucleotide substitutions per site. The genetic distances were estimated within each group and are represented in the colored circles. Each colored sector represents a certain group.

To characterize the Brazilian strains of CFAV, we chose the *E* and the *NS5* genes because they have distinct evolutionary rates and are located in different regions of the CFAV genome. The tree reconstructed using the *E* gene indicates that there are three well defined CFAV groups (i.e., Thailand, America and Australia/USA/UK) determined by high branch support values. This tree was midrooted to facilitate visualization of the topology (Figure 3A). The group Australia/USA/UK may not represent a true phylogroup because the isolates were obtained from mosquito cell lines. The remaining groups were composed of local strains/isolates and represent de facto regional strains of CFAV. This tree also shows the strains AB488425 (from Indonesia) and Macapá 02 are at the base and are highly divergent from all CFAV strains included in this phylogeny.

In addition, we also used genetic distances to illustrate the diversity of Brazilian strains. The distance among the main groups ranged from 22% to 26% (values indicated by dark braces in the tree). We also measured the diversity within each group (colored circle in Figure 3A). The diversity of the group America was estimated without the strain Macapá 02. The diversity of the Brazilian strains is also shown (blue sector in the circle of Figure 3A) and this measurement was estimated including all strains. It is important to mention that the high genetic diversity in the group of Brazilian strains was due to inclusion of the isolate Macapá 02.

Additionally, the same pattern of topology was observed in the phylogenetic tree inferred using the *NS5* region (Figure 3B). Equally, the main groups (i.e., Thailand, America and Australia/USA/UK) were supported by high posterior probabilities. The strain Macapá 02 is at the base of the clade and not within the group composed by strains from the Americas. Distances among groups ranged from 21% to 34% (values indicated by dark braces in the tree) and the diversity within each group was also measured (colored circle in Figure 3B). Note that the diversity of Brazilian strains was higher because of the inclusion of the strain Macapá 02.

### 3.3. Genetic Distances of the Brazilian Isolate Macapá 02

To better illustrate the divergence of the strain Macapá 02 from other CFAV isolates, a pairwise comparison was made (summarized in Table 2). The genetic distances between Macapá 02 and one strain of each phylogroup (Thailand, America and Australia/USA/UK) were higher than 40% (Lane 1 to 3 in Table 2) and therefore, are higher than the distance values calculated within the groups (colored circles in Figure 3). In addition, the genetic distance between Macapá 01 and Macapá 02, measured using the full length genomes, is 0.053 ± 0.002 and between Macapá 01 and NC001564 (Galveston) is 0.022 ± 0.001 (Table S1). Differences in the polyprotein between Macapá 01 and Macapá 02 are shown in Table S4.

## 4. Discussion

Here we report the first detection of CFAV in Brazil. This virus had previously been detected in mosquitoes from Puerto Rico [7], Thailand [8,9], Indonesia [10], Mexico [11] and Kenya [12]. Our findings provide evidence that this virus is also distributed in South America.

Previously, CFAV had been encountered mostly in *Aedes aegypti* [7,8,9,12] but also in *Aedes albopictus* [7], *Aedes* sp. [10] and *Culex* sp. [7]. In our study, nucleotide sequences of CFAV were detected in one pool of *Aedes aegypti* and in pools of *Culex* sp., thus suggesting that *Aedes aegypti* and *Culex* sp. are commonly infected by CFAV.

Our phylogenetic analyses revealed that one strain of CFAV (Macapá 02) was highly divergent from the other strains detected in Brazil (Macapá 01 and Macapá 04) or elsewhere (Figure 2 and Figure 3 and Table 2 and Table S4). This is the first time that divergent CFAV strains have been reported in the same country. Cook et al. [7] detected one strain of CFAV among several isolates recovered from mosquitoes *Aedes aegypti*, *Aedes albopictus* and *Culex* sp. collected in a wide geographical area of Puerto Rico. In addition, others had reported low divergence among isolates of CFAV from Mexico [11] and Thailand [9]. Therefore, our findings suggest a new strain of CFAV is circulating in Macapá, Northern Brazil, in *Culex* mosquitoes captured in the Marabaixo neighborhood.

We also report two near-complete genome sequences of CxFV. The virus had been detected in the Southeast region of Brazil, but never in the North of the country. CxFV has been found in many countries, such as Japan and Indonesia [13], Guatemala [14], Mexico [15,16,17], the USA [18,19,20,21,22], Trinidad [18], Uganda [23], Brazil [24,25], China [26,27,28], Taiwan [29], Argentina [30], Kenya [12] and Myanmar [31]. Our data corroborates the worldwide distribution of this virus.

Data from our study reinforces the previously described hypothesis about the existence of two genotypes of CxFV: One found in isolates from Asia and USA and the other in isolates from Africa, Caribbean and Latin America [18,19,23,24,28]. More recently, isolates of CxFV from Taiwan were grouped with the Africa/Caribbean/Latin America genotype [29], a classification that was also confirmed in the phylogenetic tree generated in our study.

To date, CxFV has been identified mainly in *Culex quinquefasciatus* [12,13,14,15,16,18,21,23,25,29,30] but has also been found in other species, such as *Culex pipiens* [13,19,20,26,27,28], *Culex tritaeniorhynchus* [13,29,31], *Culex restuans* [18], *Culex tarsalis* [19,20], *Culex interrogator* [17], *Culex usquatus*, *Culex* *maxi* and *Culex* *nigipalpus* [30] and also *Culex vishnui* and *Culex fuscocephala* [31]. Mosquitoes from other studies, such as *Culex* (*Culex*.) sp. [25] and *Culex* sp. [22,24,30], could not be identified to the species level, but were also positive for CxFV.

In our study, *Culex* sp. females were not identified to the species level due to difficulties in the morphological identification of mosquitoes. This technique presents the following limitations. Some critical structures of the specimens are commonly damaged or lost during their collection and/or transportation to the laboratory. In addition, identification of *Culex* sp. is laborious and requires a very experienced and well-trained entomologist. Some species of this genus can only be identified by structures of the male genitalia [36]. This is an obvious limitation in monitoring and surveillance studies of flavivirus that focus on females due to their epidemiological importance. The use of tools that allow molecular identification of the mosquitoes to the species level, such as those described by Cywinska et al. [45] and Murugan et al. [46], could be helpful to overcome the difficulties found in the morphological identification of *Culex*. sp.

Brazil is a very large country with an area of 8.5 million km^2^. Information on cISF is very limited so far and, therefore, not much can be concluded about their frequency, distribution, host range and genetic divergence in this country. However, in the last few years Brazil has been facing multiple epidemics caused by flaviviruses such as dengue virus, Zika virus and yellow fever virus [47,48,49,50]. This fact will probably lead to an increase in surveillance and monitoring studies. Since the majority of new and ongoing studies employ molecular tools, such as PCR with generic primers and nucleotide sequencing, additional cISFs may very well be detected in mosquitoes from Brazil in the near future.

In conclusion, cISFs, more specifically CxFV and CFAV, circulate in mosquitoes in the North of Brazil, Amazon region. One strain of CFAV is highly divergent from others previously described, suggesting that a novel strain of CFAV is present in this region.

## Figures and Tables

**Table 1:** Pools of mosquitoes in which near full-length genome sequences of flavivirus were obtained according to their characteristics and information about these sequences.

Sample Name	Taxonomic Category	Collection Date (Epidemiological Week)	Collection Place	Neighborhood	Near-Complete Genome Sequence
Macapá 01	*Ae. aegypti*	10/2017	P15	Central	CFAV
Macapá 02	*Culex* sp.	10/2017	P2	Marabaixo	CFAV
Macapá 04	*Culex* sp.	10/2017	P12	Central	CFAV
Macapá 05	*Culex* sp.	6/2017	P4	Marabaixo	CxFV
Macapá 06	*Culex* sp.	6/2017	P8	Marabaixo	CxFV

**Table 2:** Genetic distances between the strain Macapá 02 and other strains of CFAV according to two genome regions (*E* and *NS5*).

Comparisons	Genome Region	
	*E*	*NS5*
NC001564 (Galveston) versus Macapá 02	0.053 ± 0.008	0.049 ± 0.004
KU936054 (Aag2 Bristol) versus Macapá 02	0.050 ± 0.007	0.056 ± 0.003
AB813750 (E175-08) versus Macapá 02	0.055 ± 0.009	0.057 ± 0.004
Macapá 01 versus Macapá 02	0.049 ± 0.008	0.055 ± 0.005

Genetic distances were estimated using maximum composite likelihood method plus gamma distribution. Variance was estimated using 500 bootstrap replications. Estimations were made using MEGA 7 [44].

## Supplementary Materials

The following are available online at http://www.mdpi.com/1999-4915/10/12/666/s1, Table S1: Genetic distances of the near full-length genomes of CFAV strains obtained from this study (Macapá 01 and Macapá 02) and recovered from GenBank, Table S2: Similarity (%) of the sequence of the strain Macapá 01 with sequences of flaviviruses from GenBank, Table S3: Similarity (%) of the sequence of the strain Macapá 05 with sequences of flaviviruses from GenBank, Table S4: Polyprotein differences between Macapá 01 and Macapá 02.
