# Supplementary material for: A Novel Highly Divergent Strain of Cell Fusing Agent Virus (CFAV) in Mosquitoes from the Brazilian Amazon Region
Source: Viruses. 2018 Nov 24;10(12):666. doi: 10.3390/v10120666 (PMC6315449; doi:10.3390/v10120666)
Supplement: Supplementary file 1 [file viruses-10-00666-s001.pdf]

Table S1. Genetic distances of the near full-length genomes of CFAV strains obtained from this study (Macapá 01 and Macapá 02) and recovered from GenBank.

Comparison	Genetic distance (standard error)
Macapá 01 versus GQ165810	0.001 (±0.001)
Macapá 01 versus NC001564	0.022 (±0.001)
Macapá 02 versus GQ165810	0.052 (±0.003)
Macapá 01 versus Macapá 02	0.053 (±0.002)

Genetic distances were estimated using maximum composite likelihood method plus gamma distribution. Variance was estimated using 500 bootstrap replications. Estimations were made using Mega software version 7.0.

Table S2. Characteristics of the near full-length genome sequence (number of amino acid—aa, and nucleotide—nt) of the strain Macapá 01 and its similarity (%) with sequences of flaviviruses from GenBank according to name of the flavivirus and accession number of each sequences and genome region.

**Macapá 01**	**C**		**pr**		**M**		**E**		**NS1**		**NS2A**		**NS2B**		**NS3**		**NS4A**		**2k protein**		**NS4B**		**NS5**		**Polyprotein**	
	**128 aa**	**384 nt**	**91 aa**	**273 nt**	**56 aa**	**168 nt**	**430 aa**	**1290 nt**	**358 aa**	**1074 nt**	**239 aa**	**717 nt**	**149 aa**	**447 nt**	**587 aa**	**1761 nt**	**135 aa**	**405 nt**	**29 aa**	**69 nt**	**257 aa**	**771 nt**	**888 aa**	**2664 nt**	**3341 aa**	**10023 nt**
AeFV NC012932	18,4	32,8	24,7	41,5	37,5	51,5	33,3	44,5	63,4	60,8	61,4	63	49,7	59,3	72,1	65,7	50,4	55,8	82,6	68	58,80	61,5	78,8	69,2	59,7	58
CFAV NC001564	92,2	94	97,8	97	100	98	98,8	96,3	99,4	95,3	98,7	98	97,3	98	98,8	97,7	100	97,3	100	98,6	99,2	97,5	98,6	97,5	98,6	97
CxFV NC008604	38,3	52,7	82,1	67,7	69,7	62,8	71,9	67,7	34,6	43,9	18,4	33,4	14,4	34,7	40,2	47,5	11,9	30,9	26,1	36,2	19,8	38,8	58,2	58,7	44	48,6
DENV-2 NC001474	16,7	32,4	17,8	37	15,6	32,4	9,4	29,3	10	28	11,5	31,5	11,5	30,9	13	33	11,8	29	21,7	37,7	11,3	34,4	38,3	47,2	16,8	34
YFV NC002031	17,2	32	10,1	37,3	14,3	35,4	11,6	31,8	17,3	29	13,2	30,4	13,8	35,2	14,3	33,7	14	34,1	26,1	37,7	8,8	31,1	38	47	15,9	33,5
ZIKV NC012532	14,2	30,4	12,4	31,5	18,5	35,4	9,1	30,6	11,5	29,5	12,1	31,6	11,5	33,3	17,5	34,8	11,3	31,5	17,4	39,1	9,7	33,7	37,8	46,6	15,6	34,3

Table S3. Characteristics of the near full-length genome sequence (number of amino acid—aa, and nucleotide—nt) of the strain Macapá 05 and its similarity (%) with sequences of flaviviruses from GenBank according to name of the flavivirus and accession number of each sequences and genome region.

	**C**		**pr**		**M**		**E**		**NS1**		**NS2A**		**NS2B**		**NS3**		**NS4A**		**2k protein**		**NS4B**		**NS5**		**Polyprotein**	
**Macapá 05**	**414 nt**	**138 aa**	**252 nt**	**84 aa**	**180 nt**	**60 aa**	**1281 nt**	**427 aa**	**1179 nt**	**393 aa**	**606 nt**	**202 aa**	**438 nt**	**146 aa**	**1764 nt**	**588 aa**	**468 nt**	**156 aa**	**69 nt**	**23 aa**	**771 nt**	**257 aa**	**2667 nt**	**889 aa**	**10092 nt**	**3363 aa**
AeFV NC012932	45,1	20	42,7	19,8	50,5	36,7	47,9	32,8	52,2	35,6	31,4	18,4	31,5	18,2	46,9	42,9	35,1	12,8	43,9	27,3	36,6	19,5	57,6	58,4	43	34,6
CFAV NC001564	53,4	39,1	67	72,6	65,5	69,6	66,7	70,5	52,1	34,9	35,2	20,9	36,1	15,1	47,3	44,3	30,6	11,9	37,9	22,7	37,9	20,2	58,5	57,3	50,4	43,4
CxFV NC008604	89,4	89,9	86,1	89,3	89,4	95	87,4	94.4	88	93,9	93,4	93,6	90,9	92,5	86,6	92,9	88,7	95,5	82,6	82,6	81,1	84	88,1	93,7	87,7	92,6
DENV-2 NC001474	37,7	14	39,3	13,1	39,9	16,7	41	10,2	43,6	17,6	30,5	21,2	35,5	22,2	31,9	31,4	29,9	18,3	39,1	30,4	29,8	10,5	45,1	37,3	34,3	17,8
YFV NC002031	42,8	15,7	44,4	11	40,7	15,8	41,6	11,7	44	20,4	31,1	23,5	35,1	20,1	32,6	30,2	31,2	12,7	36,2	21,7	32,1	11,3	45,1	35,9	34,6	17,5
ZIKV NC012532	38,3	14,8	46,1	11,9	44,5	15,8	43,1	9,4	43,6	17,6	30,5	31,3	35,6	12,6	35,2	31,7	31,5	12,6	42,4	18,2	31,1	12,4	46,6	38,1	34,9	17

Table S4. Polyprotein differences between Macapá 01 and Macapá 02.

Genomic region in the CFAV polyprotein										
Capsid	pr	M	E	NS1	NS2A	NSA2B	NS3	NS4A	NS4B	NS5
T18S	I176T	I256V	A334T	K678R	L1144V	V1353I	T1542N	V2119I	M2210L	S2515T
F21S	E203G		A598S	K895R	I1183V	N1372Y	P1576S	L2135M	L2389F	G2549A
R28K			K600R	S898T	F1279S	I1382K	I1612V			V2558I
T38S			R608K	S937T	K1289R	S1396T	T1623S			I2596T
S39L			A687V	A994A		T1437I	H1659Y			S2639F
K42Q				D1001E		I1438L	V1679I			Y2644H
R54M						I1447T	R1683K			T2674A
I61V							I1701V			T2684A
S63T							T1710S			S2690L
							I1744V			E2737D
							T1833A			I2816V
							A1844S			L2824F
							R1868K			L2838S
							V1923A			V2853I
							E1995N			R2858K
							S2021N			I2860V
										A2994V
										H3004Y
										M3009I
										G3271E
										G3286A
										V3308I
